# Clinical outcomes after Descemet’s stripping endothelial keratoplasty using donor corneas from children younger than 3 years

**DOI:** 10.1111/ceo.13186

**Published:** 2018-03-30

**Authors:** Rong‐mei Peng, Yu‐xin Guo, Yuan Qiu, Jing Hong, Ge‐ge Xiao, Hong‐qiang Qu

**Affiliations:** ^1^ Department of Ophthalmology Peking University Third Hospital Beijing China; ^2^ Key Laboratory of Vision Loss and Restoration Ministry of Education Beijing China

**Keywords:** endothelial dysfunction, endothelial keratoplasty, graft contraction, paediatric donor age

## Abstract

**Importance:**

There is limited literature on paediatric donors in endothelial keratoplasty.

**Background:**

This study investigated the efficacy of and appropriate paediatric donor age for Descemet’s stripping endothelial keratoplasty (DSEK).

**Design:**

Retrospective and observational case series.

**Participants:**

Thirty‐eight consecutive patients underwent DSEK with paediatric donor corneas.

**Methods:**

The age of the donors ranged from 32 weeks gestation (premature neonate) to 3 years old. All donor consents were obtained from the parents. The causes of donor death included traffic accident, congenital heart disease and neonatal respiratory distress syndrome.

**Main Outcome Measures:**

The outcome measures included best‐corrected visual acuity, endothelial cell loss and complications.

**Results:**

Best‐corrected visual acuity at last follow‐up was >20/40 in 28 of 38 eyes (73.7%). The mean preoperative endothelial cell density of donor corneas was 4682 ± 520 cells/mm^2^. The mean endothelial cell density of grafts was 3977 ± 556 cells/mm^2^ at 18 months postoperatively. Three lenticules from premature neonate donors exhibited severe contraction postoperatively. The edge of six lenticules from donors <1‐year‐old exhibited contraction in the early postoperative period and gradually flattened spontaneously. Graft detachment occurred in one patient.

**Conclusions and Relevance:**

DSEK with paediatric donor corneas can achieve good clinical outcomes. The corneal lenticules from 1‐ to 3‐year‐ old donors are suitable for DSEK while those from donors <1‐year‐old are less suitable due to the possibility of severe postoperative graft contraction.

## Introduction

Descemet’s stripping endothelial keratoplasty (DSEK) is the preferred treatment for corneal endothelial dysfunction. However, graft dislocation and excessive endothelial cell loss (ECL) over time are the major limitations. Although the graft dislocation rate has been reduced with the advancement of surgical techniques, ECL is inevitable.

ECL is affected by storage methods, surgical stress, postoperative elevation of intraocular pressure and inflammation and subsequent intraocular procedures.^1^, ^2^, ^3^, ^4^ Therefore, the availability of high‐quantity endothelial cells (ECs) in donor tissue is important for DSEK. Theoretically, a larger graft size or higher cell density can provide higher EC counts. A graft 9 mm in diameter transfers 26% more surface area of healthy ECs than a graft 8 mm in diameter.^5^ A larger graft diameter also provides a greater reservoir of healthy ECs without significant cell loss compared with a smaller graft diameter.^6^ However, clinically, larger grafts may have adverse effects on the anterior chamber angle and the peripheral iris.

A statistically significant relationship between age and endothelial cell density (ECD) was reported in numerous previous reports.^7^ ^8^ Murphy *et al*. reported a 50.4% decrease in ECD in the foetus, an annual decrease of 17.73% in the early postnatal period between 0 and 2 years, and a 0.56% yearly reduction thereafter. The rapid decrease in cell density observed in utero and during the first 2 years of life is proportionally related to the degree of corneal growth.^9^ Elbaz *et al*. recently reported an increased rate of ECD reduction during the first 2 years of life, followed by a decreased ECD reduction rate between 2 and 5 years of age, which is similar to the reduction rate reported in adults, who have stable corneal diameters.^10^ Thus, the younger the donor, the more endothelium present in the graft.

Previous reports have suggested that paediatric donor grafts are not suitable for penetrating keratoplasty due to increased allograft reactions, smaller diameters and reduced corneal thicknesses, rendering unpredictable refractive results.^11^, ^12^, ^13^, ^14^ The elastic property also increases the difficulty in surgical manipulation.^14^ However, paediatric donor corneas have distinct advantages, such as high ECD and higher cell proliferation. Two case reports have suggested that DSEK with tissues from paediatric donors is safe with excellent outcomes.^15^, ^16^ However, before the present study, only four patients had received DSEK from donors younger than 2 years.

Using an increased number of cases, the following study investigated the outcomes of DSEK using paediatric donor grafts and the influence of donor age on graft outcomes.

## Methods

### Patients

We retrospectively analysed the outcomes of all patients undergoing DSEK with paediatric donor corneas in Peking University Third Hospital from June 2008 to December 2013. DSEK was performed on 38 eyes of 38 consecutive patients (21 males and 17 females) from 3 to 81 years old (mean age 49±22.6 years). Indications for surgery are presented in Table 1. Nine patients had glaucoma before DSEK. In this study, each patient was chosen from a wait list in sequential order when donor tissue was available in our eyebank. Before DSEK, all patients signed a consent form that was previously approved by the Institutional Review Board and Ethics Committee.

**Table 1:** Indications for corneal surgery in this study

Causes of endothelial dysfunction	No. of patients
Pseudophakic bullous keratopathy	12
Aphakic bullous keratopathy	1
Congenital endothelial dystrophy	5
Fuchs endothelial dystrophy	5
Prior DSAEK failure	5
Bullous keratopathy due to glaucoma surgery	4
Ocular trauma	3
Retinal detachment repair	2
Herpetic corneal endotheliitis	1

DSAEK, Descemet’s stripping automatic endothelial keratoplasty.

### Donor tissues

Thirty‐eight donated corneas were obtained from 19 donors ranging in age from 32 weeks gestation (WG) (premature neonates) to 3 years (mean 0.75±0.82 years) (Table 2). The causes of donor deaths included traffic accidents (*n* = 7), congenital heart diseases (*n* = 10) and neonatal respiratory distress syndrome (*n* = 2). All corneas were donated after cardiac death by the parents of the deceased donors.

**Table 2:** Details of the paediatric donors

No.	Age	Reasons for death	Time of death
1	32 weeks gestation	Neonatal respiratory distress syndrome	9 June 2008
2	5 months	Congenital heart disease	23 February 2012
3	2 years	Rupture of spleen due to traffic accident	30 May 2012
4	2 years 9 months	Intracerebral haemorrhage due to traffic accident	16 July 2012
5	10 months	Congenital heart disease	23 July 2012
6	4 months	Congenital heart disease	22 October 2012
7	3 months	Congenital heart disease	5 November 2012
8	34 weeks gestation	Neonatal respiratory distress syndrome	5 December 2012
9	6 months	Congenital heart disease	11 January 2013
10	7 months	Congenital heart disease	16 January 2013
11	2 years	Traffic accident	24 February 2013
12	2 years	Traffic accident	08 March 2013
13	3 days	Congenital heart disease	18 April 2013
14	2 months	Congenital heart disease	11 May 2013
15	1 day	Congenital heart disease	23 May 2013
16	7 months	Traffic accident	9 July 2013
17	7 months	Intracerebral haemorrhage due to traffic accident	23 September 2013
18	9 months	Traffic accident	14 October 2013
19	9 months	Congenital heart disease	20 December 2013

The redundant tissue surrounding the eyeball was carefully removed, including the conjunctiva, fascia and muscles. The eyeball was steeped in tobramycin saline solution twice for 15 min per time. Under sterile conditions, a suitable trephine was used to strip the sclera posterior cornea 3–4 mm. Then, the corneoscleral buttons were obtained and stored in Optisol‐GS (Bausch & Lomb, Irvine, CA, USA) at 4**°**C for a mean of 2.2 ± 1.2 days (range, 0.6 to 4.9 days). The ECD of all donor corneas was quantified by a certified technician at our eyebank using an EB‐3000 XYZ Eyebank specular microscope (HAI Laboratories Inc., Lexington, MA, USA) before DSEK. The preoperative cell counts were obtained using an apex digitized method with the manufacturer’s calibrations for magnification. The apices of at least 100 cells were counted from the endothelial images of each cornea. Three donor groups were divided for analysis by donor age [group 1: <0 days (*n* = 4); group 2: 0 to 1‐year‐old (*n* = 26); group 3: 1 to 3 years old (*n* = 8)].

### Surgical technique

In this study, all procedures were performed by a single experienced surgeon (JH) at the Peking University Third Hospital from July 2008 through December 2013.

#### *Donor preparation*

Before the preparation of the 38 lenticules, the preparation of two other lenticules was attempted by using a Moria automated microkeratome. However, both cuts penetrated the centre because the paediatric corneas were small, thin and soft. Therefore, all 38 lenticules were prepared by deep manual dissection. The anterior lamella of 70% to 80% stromal thickness was bluntly dissected. The posterior lamellar tissue was punched from the endothelial side with an 8.0‐mm trephine. The donor corneal lenticules were then immediately covered by viscoelastic (Viscoat, Alcon Surgical, Inc., Ft. Worth, TX, USA) until transplantation.

#### *Recipients*

According to the status of the recipient eyes, at the time of DSEK, some patients underwent combined surgeries, such as phacoemulsification cataract extraction and posterior chamber intraocular lens (PC‐IOL) implantation (*n* = 4), removal of an anterior chamber (AC)‐IOL and placement of an IOL in the sulcus with suture fixation (*n* = 6), and suture fixation of a PC‐IOL in aphakic eyes (*n* = 2). A 4‐mm temporal sclera tunnel incision was made. The AC was filled with viscoelastic. Further paracentesis wounds were made at the limbus at the 6‐o’clock position and each side of the main incision. Descemet’s membrane and the dysfunctional endothelium were scored with a blunt‐tip hook using the epithelial mark as a guide, stripped off using a stripper and removed with forceps. An AC irrigator was inserted via the left paracentesis beside the main incision.

#### *Donor insertion*

A donor lenticule was gently transferred onto the donor well of a Busin glide (Moria, Doylestown, PA, USA). The lenticule was pulled into the glide opening with forceps by holding the leading stromal edge, and a 10–0 nylon suture was placed to pull the lenticule into the AC. The suture was knotted and inserted through the main incision into the AC and then pulled from the 6‐o’clock paracentesis. The AC viscoelastic was removed. The glide was inverted and positioned at the entrance of the corneal tunnel. The lenticule was pulled into the AC by the suture via the 6‐o’clock paracentesis. The suture was then removed. The wounds were closed with sutures. The grafts were pressed up against the recipient cornea with filtered air. The graft position was verified with the slit lamp on the microscope. Interface fluid was squeezed out.

### Postoperative follow‐up

The clinical outcome of DSEK was assessed by best‐corrected visual acuity (BCVA), ECD and complications at 1, 3, 6 months postoperatively and every 6 months thereafter. Graft attachment and contraction were assessed with anterior segment optical coherence tomography (AS‐OCT; Carl Zeiss Meditec, Dublin, CA, USA). ECD was measured by laser confocal scanning microscopy (HRT‐3: Heidelberg Engineering, Heidelberg, Germany). The same certified ophthalmic technician performed all postoperative testing of patients using the same microscope.

### Statistical analysis

Statistical analysis was performed with SPSS v16.0 (SPSS, Inc., Chicago, IL, USA). The main outcome measure was ECD at different times. Donor age and ECD associated with different donor age groups were assessed using analysis of variance (ANOVA). All tests were 2‐tailed, and *P* < 0.05 was considered statistically significant.

## Results

### Outcomes and common complications

The average follow‐up time was 26 months (range 18–45 months). BCVA at the last follow‐up was >20/40 for 28 of 38 patients (73.7%). In some patients, pre‐existing conditions limited visual acuity and included advanced glaucoma (*n* = 4), amblyopia (*n* = 5), macular scar (*n* = 2) and optic nerve dystrophy (*n* = 1) due to trauma. Postoperative refractive astigmatism was within three diopters (D) in all patients. Myopic shift was 0.85 ± 1.34 D. The mean corneal astigmatism was 2.47 ± 1.50 D. The mean true‐net power was 41.2 ± 1.0 D. The mean thickness of the central grafts was 110 ± 65 μm at the last follow‐up (range, 22–212 μm; interval, ≤50 μm [*n* = 3], 50–100 μm [*n* = 18], 100–150 μm [*n* = 9], >150 μm [*n* = 8]).

Graft dislocation (donor age 3 months) occurred on the first postoperative day in a buphthalmic eye. *Re*‐bubbling was performed, which resulted in a microfracture. However, the graft remained attached at a later follow‐up.

Postoperatively, the intraocular pressure of four eyes was elevated and was controlled by medical treatment. Of these, three eyes had a history of glaucoma and one eye newly developed glaucoma following DSEK. No graft rejection was observed.

### Graft contraction

**Figure 1:**
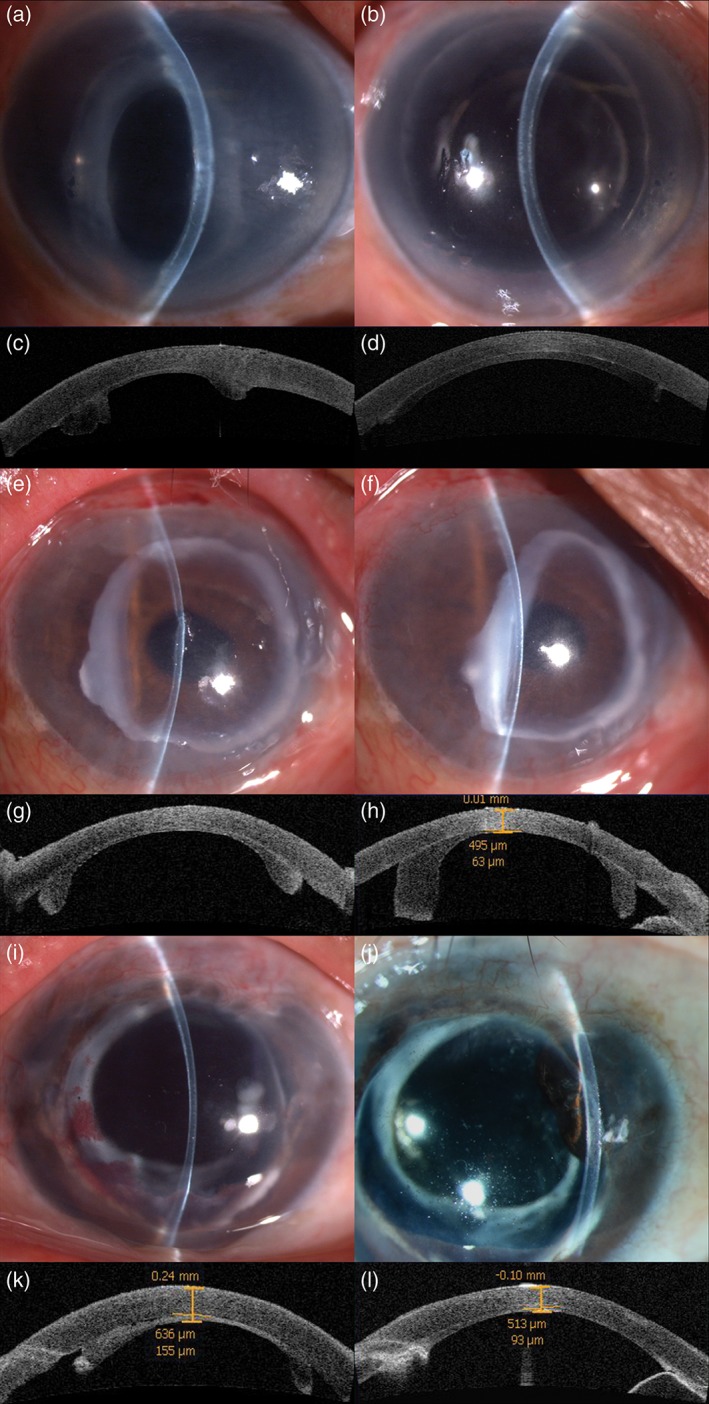
Anterior segment (AS) photograms and AS‐OCT of three patients with graft contraction. (a, c) One patient underwent DSEK with a lenticule from a donor at 32 weeks gestation (WG). The periphery of the graft was contracted, thickened and opaque 3 months after surgery. The cornea of the recipient with the non‐contracted graft was transparent, and the remainder of the cornea was oedematous and opaque. (b, d) The contracted graft was replaced by an adult graft. The new graft attached well, and the cornea was transparent. (e–h) One patient underwent DSEK with a lenticule from a donor at 34 WG; 7 days (e, g) and 6 months (f, h) postoperatively. The graft contained corneal limbal tissue. The graft attached well in the early period. However, the periphery of the graft contracted. The area of the cornea over the pupil was transparent, and the patient was satisfied with the visual acuity. Therefore, the graft was retained. (i–l) One patient underwent DSEK with a lenticule from the preceding donor. In contrast to the previous graft, the periphery of this graft was contracted in the early postoperative period and became flattened at 3 months.

**Figure 2:**
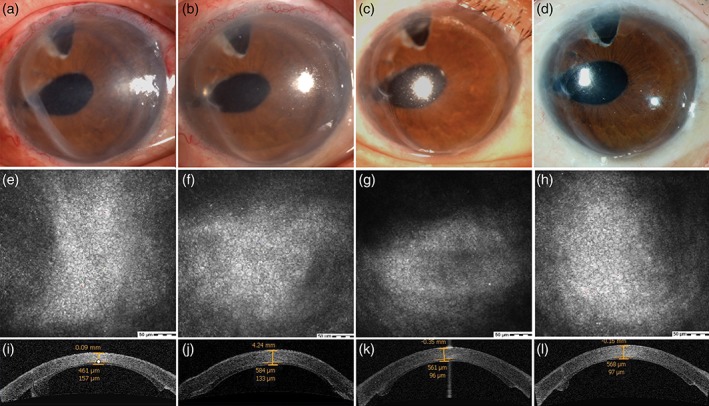
One graft from a 3‐month‐old donor showed temporal contracture during the first postoperative week and was flattened at the 3‐month follow‐up. (First row) The infratemporal graft was contracted 1 month after surgery. The central thickness of the graft was 157 μm, with an ECD of 3289 cells/mm^2^_._ (Second row) The graft became flattened at 3 months. The central graft thickness was 133 μm, with an ECD of 3217 cells/mm^2^. (Third row) The central graft thickness decreased to 96 μm after 6 months. The ECD was high at 4157 cells/mm^2^. (Fourth row) The central graft thickness was 97 μm after 1 year. The ECD was up to 4945 cells/mm^2^.

Graft contraction was a particular complication in this study and is not commonly noted with adult grafts. Starting from postoperative week one, various degrees of contraction were observed in nine grafts. The donor ages were 32 WG (*n* = 2) and 34 WG (*n* = 2), 1 day (*n* = 1), 3 days (*n* = 1), 3 months (*n* = 1) and 7 months (*n* = 2). All donors were <1‐year‐old. Three grafts from premature neonate donors (two at 32 WG, one at 34 WG) developed full peripheral circular contraction which progressed daily. Of these, two grafts had to be replaced with adult grafts at 10 days and 3 months postoperatively because the patients experienced worsening visual acuity (Fig. 1a–d). The other graft from a donor at 34 WG was not replaced because the 76‐year‐old patient was satisfied with the visual acuity (20/60) (Fig. 1e–h). The other six grafts exhibited partial peripheral contraction, which gradually flattened at 2‐ to 6‐months of follow‐up, including one graft from a donor at 34 WG (Fig. 1i–l). Figure 2 displays the resolution of one partial graft contraction from a 3‐month‐old donor. Partial temporal contraction noted during the first postoperative week flattened spontaneously at the 3‐month follow‐up.

### EC loss

**Figure 3:**
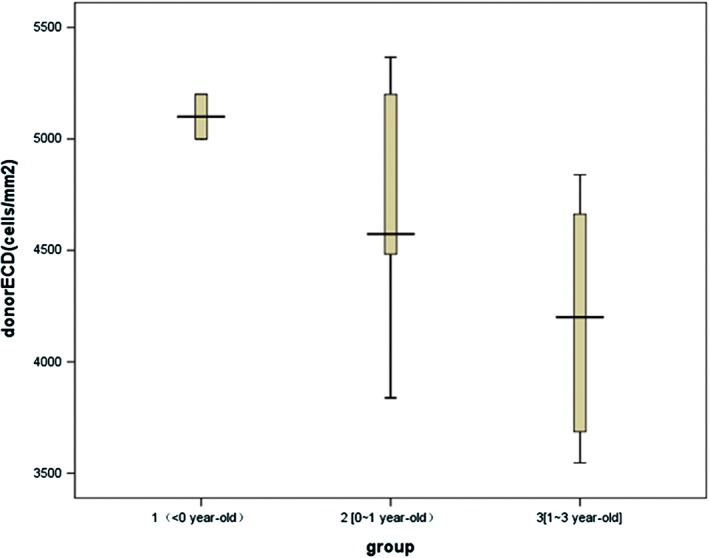
Box plots of ECDs of grafts from donors at different ages. Group 1: premature infants (<0 years); group 2: 0 to 1 years old; group 3: 1 to 3 years old. A significantly lower ECD was noted in group 3 (*P* < 0.05) than in the other groups.

**Figure 4:**
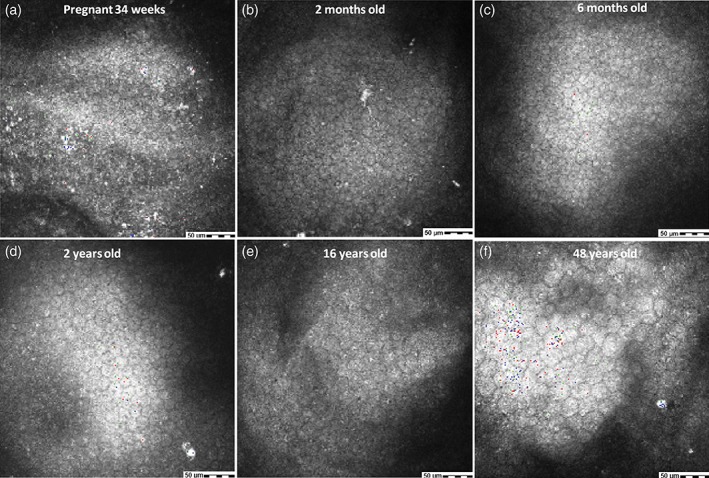
Morphological changes in endothelial cells of donor grafts at different ages assessed by HRT‐III staining at 3 months postoperatively.

The ECD of the donor corneas was 3407–5366 cells/mm^2^ (4682 ± 520 cells/mm^2^). The mean ECD values for groups 1, 2 and 3 were 5100 ± 115, 4771 ± 450, and 4186 ± 542 cells/mm^2^, respectively (Fig. 3). Groups 1 and 2, in which all donors were younger than 1 year, did not differ in ECD. However, the ECD was reduced in group 3 compared with that in the other groups (*P* < 0.05), suggesting a significant decrease in donor ECD after age 1 year. In association with the reduction in ECD, the size of ECs increased as donor age increased (Fig. 4).

The mean ECD of the grafts was 3977 ± 556 cells/mm^2^ (*n* = 33; range, 2530–4945 cells/mm^2^) at 18 months postoperatively, representing a mean EC loss postoperatively of 14.0 ± 11.5% (range, −6.68–47.56%). The mean EC losses for groups 1, 2 and 3 were 16.9% (*n* = 1), 13.3±8.5% (*n* = 25), and 16.3 ± 20.1% (*n* = 7), respectively. The EC density was increased in 3 cases (EC loss rate: −3.37% [group 2], −5.07% [group 3] and −7.89% [group 3]). ECD data could not be collected for five patients because two received replacement grafts as mentioned previously, one died 2 months after surgery, and the remaining two declined the ECD assay. No significant differences were noted between groups 1 to 3.

In addition, the ECD in seven patients increased over time (Table 3), as demonstrated in one patient in Figure 2. All grafts were from donors <2 years old. The time of ECD increase was 3 (*n* = 3), 6 (*n* = 2) and 12 (*n* = 2) months, postoperatively.

**Table 3:** Details of the seven patients with increased endothelial cell density (ECD) postoperatively

Patient No.	Donor age	ECD of donor graft (cells/mm^2^)	ECD of graft at follow‐up, months postoperatively(cells/mm^2^)				
			1	3	6	12	18
1	5 months	3548	3451	3529	3418	3859	3828
2	2 years	4335	4357	4171	4180	4481	4516
3	4 months	5200	3147	2722	3473	3923	3896
4	3 months	5123	3289	3217	4157	4945	4973
5	32 WG (premature neonate)	5000	3518	3970	4134	4217	4155
6	6 months	4519	3432	3917	3995	4054	4019
7	7 months	5200	3592	3986	4176	4032	4100

WG, weeks gestation.

## Discussion

In this study, using corneas from paediatric donors, BCVA at the last follow‐up was >20/40 in 28 of 38 patients (73.7%). DSEK with paediatric donor corneas can achieve good clinical outcomes. The lenticules from donors aged 1 to 3 years are ideal, while those from donors <1‐year‐old contract easily and are not suitable for DSEK.

### EC loss

DSEK is an effective treatment for endothelial dysfunction. Theoretically, if a graft harbours more ECs preoperatively, the graft will have a better survival advantage. Given that the number of ECs decreases with age, grafts from paediatric donors with a higher ECD may provide such a benefit.

In this study, the mean preoperative ECD in paediatric donors was 4576 ± 617 cells/mm^2^ (range 3407–5366 cells/mm^2^), whereas the value for adult donor corneas is typically from 2270 to 4209 cells/mm^2^.^5^, ^17^, ^18^ In previous reports, ECL ranged from 13.7% to 54.2%.^19^, ^20^ Previous studies on paediatric donor corneas (3–5 years old) used for DSEK reported that the average endothelial cell loss was 41.4 ± 3.7%.^21^ The factors affecting ECL include the type of graft insertion forceps used,^22^ whether a combined procedure was performed, the occurrence of complications, the duration of graft storage, the surgeon’s technique^23^, ^24^ and even the width of the incision.^3^ In our hospital, a previous report demonstrated an ECL of 39% at 12 months post‐surgery.^25^ In this study, the mean ECD of the grafts was 3977 ± 556 cells/mm^2^ at 18 months, representing a mean ECL of 14.0 ± 11.5%. This reduced ECL rate may contribute to improved graft longevity, which remains to be verified in future studies. In our study, one graft underwent initial reverse implantation, which was corrected during surgery, and re‐bubbling had to be performed twice, postoperatively. The ECD of this graft was 3171 cells/mm^2^ at 18 months, equating to a 41% ECL following DSEK. In our experience, grafts that undergo this operative manipulation likely become dysfunctional in 18 months. However, because the donor age was 3 months and the preoperative ECD was 5366 cells/mm^2^, the graft survived well, and the patient achieved a BCVA of 20/50 at 30 months (last follow‐up).

We identified no significant difference in the ECL rate in grafts between all donor groups in this study. This result illustrates a similar vitality of the ECs in donors <3 years old. Although the ECD of grafts from 1‐ to 3‐year‐old donors was significantly lower, the ECL rate was not significantly increased compared with that in <1‐year‐old donors. Grafts from 1‐ to 3‐year‐old donors exhibited a reduced number of ECs, but this reduction did not seem to be a disadvantage overall.

In addition, the number of ECs increased over time in seven patients. Such a result may indicate that the ECs may multiply in grafts from donors <3 years old. *In vivo* human corneal endothelial cells are thought to be arrested at the G1 phase of the cell cycle; thus, proliferation is inhibited.^26^ A purely medical treatment for corneal endothelial disease has been sought for quite some time, and several interesting agents have been explored for this purpose, such as EDTA,^27^ EGF, PDGE, FGF‐2,^28^ siRNA of Connexin 43,^29^ R‐spondin 1^30^ and ROCK inhibitor.^31^ However, these agents have not yet been introduced into the clinical setting. These findings indicate the possibility of human endothelial cell proliferation in vivo, supporting the study hypothesis. However, this hypothesis requires further investigation.

### Graft dislocation

In this study, one graft dislocation occurred in an eye with congenital glaucoma. The diameter of the recipient cornea was 14 mm, and the AC was deep. The posterior corneal surface topography was irregular because the patient had undergone two DSAEK procedures with adult donor corneas. The first graft failed 2 years postoperatively, whereas the second graft failed 6 months postoperatively. The corneal curvature was steeper in the buphthalmic eye. In addition, the patient had previously undergone multiple surgeries for glaucoma. However, overall, in this study, the graft dislocation rate was lower than that reported previously in our hospital (2.6% *vs.* 19%).^25^ The grafts may have been soft and highly plastic and thus conformed to the posterior corneal surface curvature better after the injection of air.

### Graft contraction

In this study, graft contraction was a common complication (9/38) that is not commonly observed in eyes implanted with adult grafts. All nine grafts with contraction were from donors <1 year old. Three of these were from premature neonates who died of respiratory distress syndrome, and the contractions were very severe. Two grafts had to be replaced with adult grafts. Six grafts exhibited partial contraction in the early postoperative period but flattened spontaneously at 2 and 6 months postoperatively. This finding may be explained as follows. First, the development of paediatric donor corneas may be incomplete, and tissue elasticity is greater. An infant’s eyeball at birth is approximately 80% of the adult size.^32^, ^33^ The postnatal sclera and cornea are somewhat distensible, gradually becoming more rigid during the first 2 years of life. The eyes of premature neonates are even less developed than the eyes of term neonates. Given their elastic nature, such grafts naturally contract and separate from the posterior cornea without air support. Second, because the neonate cornea diameter is smaller than that in adults, a small rim of scleral tissue is likely included in the donor graft during lenticule preparation. As shown clearly in Figure 1, the grafts in case 2 (e–h) and case 3 (i–l) were from the same donor. However, the degrees of graft contraction differed. The anterior segment photographs revealed that the graft in case 2 contained more scleral tissue (the white rim), and the sclera was curled inward, leading to graft edge detachment.

This study is subject to the limitation of the small sample size in the 1–3‐year‐old group. But it can be readily concluded that DSEK with donor corneas from children <3 years old can achieve good clinical outcomes. The corneal lenticules from 1‐ to 3‐year‐ old donors are suitable for DSEK while those from donors <1‐year‐old are less suitable due to the possibility of severe postoperative graft contraction.
